# Fortieth Annual Meeting Miss. Valley Dental Association

**Published:** 1884-04

**Authors:** E. G. Betty


### PROCEEDINGS.

### Fortieth Annual Meeting of the Mississippi Valley Dental Association---Proceedings and Discussions.

REPORTED BY E. G. BETTY, D.D.S.

This Association convened for the fortieth time, in the lecture-room of the Ohio Dental College, Wednesday morning, March 5, 1884, at ten o'clock a. m.

President Wright called the meeting to order, when prayer was offered by Prof. Taft.

In the absence of the Recording Secretary, W. H. Cameron, Mr. Otis Cameron was appointed Secretary *pro tem.*, and very efficiently and acceptably performed the duties of the office.

The minutes of the thirty-ninth annual meeting were read, and no objections being offered, stood approved.

Dr. J. Taft, Chairman of the Executive Committee, presented the following programme for the consideration of the members :

I. Artificial crowns upon roots; best method of adapting them.

II. What means are best adapted for maintaining the teeth and mouth in a healthy condition?

III. What progress has been made in arresting and preventing decay of the teeth, in the last five years?

IV. In what respect, if any, are decayed teeth better filled now than ten years ago?

V. What valuable medicinal agents have been introduced into dental practice in the last three years? For what purpose is each one used?

VI. What improvements, if any, have been made in artificial dentures, during the last five years; and what further improvements are desirable?

On motion of Dr. H. A. Smith, it was adopted.

Dr. J. Taft moved that the hours of the sessions be fixed at from 9 A. M. to 12 M., and from 2 P. M. to 5:30 P. M., omitting the evening session as heretofore held.

Dr. Berry said that, in the old times agone, the Society had always held evening sessions, and that the members took advantage of it to improve their minds with professional business. It is not proper that we go back on the good record, and waste the three additional hours the evening gives us. He objected most decidedly to doing away with the evening session, as he, for one, had no desire to visit the resorts "*Over the Rhine.* [Applause.]

Dr. Van Antwerp thought that somebody else wanted to see the "dog show" besides Dr. Berry. [Laughter.]

Dr. J. Taft—There seems to me to be no reason for continuing the night sessions. The six hours and a half allotted for work during the day is ample time in which to accomplish a day's work, and, in my opinion, we need the relaxation the evening gives us to recover from the exhaustion many experience who have faithfully attended and taken part in the day's discussions.

Question being called, Dr. Taft's motion was carried unanimously.

On motion of Dr. Betty, a recess of five minutes was taken, for the purpose of enabling the Treasurer to collect dues.

Dr. Hunter, of the Committee on Membership, proposed Dr. F. M. Williams, of Colorado, for membership.

On motion of Dr. Betty, the gentleman was elected by Secretary's ballot.

The question of disposition of such essays, addresses, or papers, that might be presented before the Society, was broached by Dr. Berry, who advocated a division of them between the REGISTER and the *Ohio State Journal.*

DR. ROSE moved that the reports and essays be referred to the Committee on Publication to be disposed of at its discretion.

DR. BETTY said that, though the Society had the right of possession of such essays as might be read before it, it had *no* right to the *reports* being made, as they were made by enterprising journals, and were private property. Though we all respect Dr. Watt very much, it must not be forgotten that the REGISTER has been the recognized organ of the Society for many years past, and it would seem that, if any journal had a claim, it would be the REGISTER.

Drs. Hunter and H. A. Smith both spoke in the same vein; and, on motion of the latter, the Committee on Publication was instructed to furnish duplicates to both the REGISTER and the *Ohio State Journal* for simultaneous publication, to the satisfaction of all.

No further miscellaneous business presenting itself, the first subject on the programme was announced for discussion.

Dr. G. W. Smith then read a paper bearing upon it.

There being no other papers, and the hour of adjournment nearly arrived, Dr. Taft moved that the session adjourn, and at 2 o'clock a special order of business be made, in order to give Dr. Howe ample time to illustrate his method of attaching artificial crowns. Carried.

### FIRST DAY—AFTERNOON SESSION.

Upon assembling, the members were not only entertained, but instructed, by Dr. Howe's descriptions and methods; embracing, in regular order of development, the wooden pivot, plate tooth with metallic pivot, and porcelain crown attached with screw operated through an opening in the crown and into the root by a slot in the screw-head. He then went on to describe his particular method of attaching crowns by systems of screw-posts, crowns with four long pins, locking with cement, etc.

Dr. H. A. Smith—In those cases where the canal is very much enlarged, and the root wasted away, it becomes a difficult matter to fasten the post securely. To remedy this, I am in the habit of filling the root with lead, first introducing the post, and filling around it; then unscrewing it to adjust the crown, as the bending of the post produces too much strain.

Dr. Howe—The post can be bent in any direction to suit the case without causing a strain upon the attachment.

Dr. H. A. Smith—According to my idea, the post must be bent to bring it into alignment, before it is finally fastened.

Dr. Howe--By using the chuck, the post can be bent, even after it is fast, without any stress.

Dr. H. A. Smith—It takes a good deal of force to bend it, and there is, in consequence, great liability of changing the position. The chief advantage needed is facility in bringing the post into alignment, and many failures result because it cannot be done to suit.

Dr. Hunter—It is not necessary for me to say much, after Dr. Howe's able descriptions. I have had a good deal to do with mounting artificial crowns, and consider his method superior to all, after an experience of one year, during which I mounted some ten or twelve cases, all of them most satisfactory. I consider the system perfect, stronger than restorations made with gold, and the complete control had over the several steps in the system, constitute one of its great beauties.

Dr. J. Taft--I have not used this, or other methods, to any great extent; but am well satisfied that if all the steps are carefully executed, good results will be obtained, probably more than in other methods, and recommend it highly. The great trouble with operators is, that they proceed too hastily in their endeavors to accomplish the best results. There are difficulties in the method under consideration, that the novice would not comprehend; so it is important, then, that every one must have patience in working out the minute details before he can secure a satisfactory result. Another point: in cases where decay has been extreme, too much is attempted, and the result does not meet the anticipation, though the results are sometimes good. Where there is a predisposition to irritation, too much should neither be attempted nor expected, though the operation may be made more in the way of an experiment. If the patient is healthy and robust, almost anything may be attempted and crowned with success, no matter how much irritation may have been made. In such a case, where the gum rests upon a sharp edge of the root, there may be more or less irritation, tumefaction, and formation of pus; yet, if proper attention is paid to the treatment of it, the crown may be retained without much or any discomfort, and be a success. We must not be too sanguine of obtaining a perfect result in every operation we attempt. The surrounding conditions are important factors, having a more or less direct bearing upon the success or failure of the result that is reached. If the crown itself should project, or the fastening cement either, it furnishes a pocket for the retention of irritating substances that, if not remedied, will defeat success.

Dr. H. A. Smith—I do not wish to be understood as meaning hopeless or doubtful cases, as it is evident that there must be some hope to go upon. The point I wish to make has no pathological bearing; it merely concerns those hollowed-out cases where there is not much chance for attachment.

Dr. Howe—The cases Dr. Smith refers to, no doubt, are those that need extracting.

Dr. G. W. Smith—In the last year I have not met with a case that could not be treated and successfully crowned. The Howe method takes the place of a partial plate, and is, probably, the best and most feasible in use.

Dr. Sillito—I had a case that had to be treated for three or four months before it could be crowned, and, after a lapse of nine months, remains good, so far as I have heard.

Dr. J. Taft—I did not tell all in my last remarks. If the gum overlaps the edges of the root, part of the treatment is to crowd it away with cotton, in order that better vision and more space may be obtained by fully exposing the root, so that the crown may be adjusted with more precision, and better finished. Another thing: It is easy to crowd the gum away; but, in many cases, there is danger of setting up a diseased action that may go on for some time, and, possibly, defeat success; but this must be guarded against. I would like to say right here, Mr. Chairman, that it is commonly supposed that it is only those who know something, should do the talking, and I would suggest that those who know nothing, should be called on to say *something*. [Laughter.]

THE PRESIDENT—Shall I call upon them?

DR. TAFT—Yes, sir. About ten years ago Dr. Corydon Palmer carved out of a walrus' tusk a crown so good that no one could detect its artificiality; this was for, and still is, in his own mouth, attached with screws.

DR. BERRY—Though I have very little, or nothing to say upon the subject, I apprehend that trouble may be expected if crowns are set upon diseased roots. One case in point I may mention: Two central incisors were mounted, and next day the patient's face was swelled to an alarming extent, and considerable pain accompanied. However, the patient went through it bravely; ten years elapsed, when one of the roots was extracted, the other remaining for some time longer. So, you see, if the patient has confidence in the dentist, the latter may make a mistake, and the patient be induced to undergo the consequences, and a good result finally take place.

THE CHAIR—We should like to hear from Dr. Jennings upon this subject.

DR. JENNINGS—The topic is in good hands, and I enjoy listening.

DR. HUNTER—Think it a bad thing for Dr. Jennings to absorb all, and not give in return, as it was setting a bad example to the bashful members.

DR JENNINGS—Dr. Hunter once said he was like the Dutchman who would eat, drink, and pay for nothing. [Laughter.]

DR. SAGE—Mr. President, I think it would be a good idea to ask the members to tell us what they regard as the *worst* methods for mounting crowns. The Bonwill, when set with amalgam, loosens sooner or later, because the pin becomes amalgamated, and finally breaks off, and in a short time, too, sometimes not longer than a few weeks. The Darby method has the advantage of economizing time, and can be completed in the one sitting, and the results are reliable. I do not like the use of oxyphosphate so well as lead or gold, as there is too much recoil; prefer gutta percha in chloroform, or the oxy-chloride, but the latter washes out.

Dr. G. W. Smith—The gentlemen have not caught my idea; the fossiline and surgeons' cotton can be carried clear to the apex.

Dr. Van Antwerp—As I did not hear Dr. Howe's descriptions I would like to ask him if he illustrated the cutting of the circle on the end of the root?

Dr. Howe—Yes, I did.

Dr. Van Antwerp—My trouble has been that a small space would be left there, and that the crown would loosen in consequence.

On motion the subject was passed and the second announced.

Dr. Berry—Owing to the extreme modesty of my brethren about opening the discussion I feel obliged to do it myself, in order to preserve the reputation of the profession. Whatever will best conserve the health of the body generally will act equally upon the teeth, so I would recommend good food, plenty of exercise, baths, recreation, amusement, etc. I would avoid the use of tobacco, alcohol, tea, and coffee, and let food and drink be of the simplest nature possible. Absolute cleanliness is of course to be observed; the dentist consulted at least twice a year until the age of forty years, after which time the necessity is not so urgent. If all of these measures are properly attended to we will then be doing the best we can to maintain healthy mouths and teeth.

A paper on "Phosphate of Lime and the Teeth" was then read by Dr. Wright. (It appears elsewhere in this issue of the Register).

Dr. Jay—I am very much pleased with Dr. Wright's paper, and would like very much to hear more upon the subject from other members.

Dr. Rawls—There are very few who know how to administer complementary foods. The tissues of the body will be weaker or stronger in perfect accordance with the supplies of materials needed in the food consumed. In addition to this, there must be a capability of the system to appropriate and assimilate the materials offered in the food, for, if this ability does not exist, it is manifest that there will be no result. In the pregnant woman all her systemic energy is required to support herself, and as the child is, at the time, part and parcel of the mother, it will receive all the needed supplies if the mother gets the proper nutriment, together with the complementary diet, if her system is in condition to digest and assimilate it. We cannot expect one given to hard labor during pregnancy to give birth to a perfectly healthy child. It is a prime necessity that she have exercise for both body and mind, that she be kept in a healthy condition.

In childhood the teeth should be *used* in the full sense of the word, and the child should be taught so to do; in fact, all parts of the body should be exercised equally. If exercise is not resorted to, to increase the activity of the circulation, the complementary substances will not be taken up but become effete and thrown out, they having surcharged the body with those elements. All of the complementary foods and medicines (if you choose to call them so) have their place, but a great many are pushed on the patient when they already are fully supplied, if they use it properly by exercise, light, air, etc. Again, I say, the teeth must be *used* thoroughly to bring about development.

Dr. H. A. Smith—Some very crude statements have been made during the discussion of this subject. Some races of men live on a vegetable diet, others on meat, and still others on that almost altogether albuminous, yet they all have equally good teeth; why is it? In a paper in the Agricultural Bureau the author says there is less albumen in American wheat than in the English, the percentages being: American, 11.55; English, 13.; Pacific Coast from 8. to 10. The hexagonal cells of the grain contain the starchy elements; in the six layers on the outside reside the albuminous elements. The English prefer the starchy parts, while the Americans think the outer layers are the most important, as they also contain the phosphates. In macaroni there is 25 per cent. of albumen, and, in consequence, the eater of it needs no meat. Are his teeth any better or any worse than those of any other nation? As the chick develops the shell becomes thinner, its substance going to supply the bones and feathers. In treating pregnant women the whole system, bones, and teeth appropriate the phosphates. In my own family myself and elder son have eaten brown bread for twelve or thirteen years, yet his teeth are the poorest of the four children.

Dr. G. W. Smith—What do you think of a milk diet?

Dr. H. A. Smith—Milk is a most excellent article of diet, and it contains more phosphates than cereals or meat. In the growing child no phosphates are eliminated, because the demand for them equals the supply; but when the bones *are* grown the excess is thrown out as useless.

Dr. Van Antwerp—When he was making his dictionary, Dr. Johnson said that oats were fed to horses in England and to men in Scotland. Dr. Rawls hit the key note of the whole thing when he stated that diet was of secondary importance to exercise, light, and air. The Highlanders of Scotland exercise outdoors a great deal, and they possess most excellent teeth.

Dr. Wright—How can you give the fœtus outdoor exercise?

Dr. Van Antwerp—If the mother is so assisted that she will not be run down or worked to death, nature itself will supply both her and the child, especially if she have plenty of outdoor exercise.

Dr. H. A. Smith—There are many theories about the qualities of the waters we drink. A gentleman once said to me that there are more bandy-legged children near Glasgow and in the Orkney Islands than he had seen anywhere else. I have very little faith in the efficacy of lacto-phosphate in increasing the quality of the teeth. The mother herself will select such food as she most needs, and which is best for the fœtus.

Dr. Wright—Dr. Cushing said that if the mother will not or cannot live in the manner Drs. Rawls and Van Antwerp think she should, that the preparations of phosphates will be absorbed and assimilated when that in the usual food will not. The Tuscans do not wash themselves, live in dirt, and eat macaroni. The Germans in the Konigstuhl have teeth that are better than in other parts of Germany. In Berlin the teeth are softer than elsewhere; and I believe, it is said, the egg-shells are thinner, too. In localities where the teeth are better the volcanic nature of the soil was thought to be the cause of its richness in the mineral constituents.

Dr. H. A. Smith—The point I make regarding the grain of wheat is to show the different value placed upon the different parts of it, the inner and outer. The English prize the starchy parts, and hold our wheat in great estimation because it contains so much, while we value their wheat because it contains more phosphates and albumen.

Dr. Wright—The value of the wheat is in proportion to the ingredients it contains that we wish to get out of it.

Dr. Sage—Dr. Garretson says that the weakness of the mother's is not due to the absorption of phosphates for the fœtus.

Dr. Osmond—Where there is a loss of appetite the administration of phosphates will do no good, for the reason that they will not be appropriated. I alternate the administration with an interval of a week, as that time seems to be needed to rest, then resume again for another week.

### SECOND DAY—Morning Session.

The meeting was called to order by President Wright, and the minutes of first day's session read and approved. Dr. J. Taft announced the death of Dr. J. B. Kidd, of Lexington, Ky., and moved that a committee of three be appointed to draught suitable resolutions, Dr. A. O. Rawls to be Chairman. The President appointed Drs. Hoff and Betty, as they are members of the class Dr. Kidd graduated in. Dr. Taft also stated that he thought this Association ought to take some action upon the death of Prof. Buckingham, and would like the members to say what they thought about it. Dr. G. W. Smith said that he studied under Prof. Buckingham, and graduated at his college, and that the professor had, by his kindness and attention to the students, endeared himself to all. Dr. Howe said that he also had graduated under Dr. Buckingham, and was present at a meeting in Philadelphia where resolutions were passed subsequent to the doctor's demise, and observed the great unanimity of sentiment among the members. Dr. J. Taft moved that a committee of three be appointed, of which Dr. Howe was to act as Chairman. Carried, the President adding Drs. G. W. Smith and J. Taft to it.

The discussion of the second subject was then resumed.

Dr. J. Taft—I was very much interested in Dr. Wright's paper and the discussion of it, and think that it is very suggestive. I would, however, like to say a few words about the care of the teeth in daily practice, for I think there are some points that should not be neglected. A great many of the remarks by the members yesterday had reference more to the development of the teeth. Now, then, after the teeth are erupted and in place, what is the best thing for, or what are the best means of, maintaining them in the best possible condition? It is too frequently the habit of most practitioners to merely do what the patient desires and without any reference to other conditions that stand in need of intelligent treatment, generally filling the tooth or teeth and dismissing the patient without further say. Some times teeth are filled that are surrounded and often covered with accumulations of tartar, which is not removed as it should be. The first point to be attended to is the putting of the mouth in as healthy a condition as possible, as in the proper order of proceedure a healthy condition should be established first. All accumulations and impurities of whatever kind should be thoroughly removed. Then the thought suggests itself, what are the best means to keep the mouth so? Impress upon the mind of the patient that he or she is equally responsible for the condition of the mouth and teeth, and that it is a personal duty to give them the necessary attention, in order that the dentist may have assistance in guarding against the advent of disease. The patient must be taught *how* to preserve the most thorough cleanliness. When all else is favorable and healthy, filling is the best way of conserving the teeth, ordinary fillings saving them in many cases as well as fine ones, though all should be as perfect as skill can make them.

Dr. Osmond—I wish to endorse all that Dr. Taft has so ably said upon this subject, and will add that I play the scavenger first, disagreeable as it frequently is. I then direct powder to be used at night, as I regard that as the best time for using the brush. At the first sitting I do not extract any roots, for, after the mouth is clean one or more good roots may be found suitable for carrying artificial crowns.

Dr. H. A. Smith—All agree with Drs. Taft and Osmond in regard to the cleanliness of the mouth. If one cannot write a Latin prescription for necessary remedies use listerine instead, as it is a very good thing. In the mouth are found numerous bacteriæ which cleanliness and the use of some suitable remedy will remove. I would like to call attention to the history of the discovery of bacteriæ. On September 14, 1683, Anthony Van Lenkenhoek gave notice to the F. R. S's., that he saw under the microscope bacteriæ in matter scraped from his own teeth, and of which he recognized several varieties. He found that scrubbing the teeth and the use of hot coffee destroyed them. It is true that heat destroys them; but, the use of the coffee must be persisted in, and at a temperature of at least 100° Fahr. An antiseptic mouth wash is undoubtedly the best thing for the purpose, and I know of none superior to listerine.

Dr. Berry--I think that hot coffee is a bad dose for the patient's stomach. A physician that I am acquainted with, and said to be a good microscopist, found bacteriæ in his saliva, showed it to his wife, who said there was none in hers. An examination with the microscope showed that there was, but not in such numbers, as the physician's mouth was in a bad condition and pyorrhœa alveolaris present besides, while his wife's mouth is a clean one. This case shows the difference between healthy and diseased mouths.

Dr. G. W. Smith--We can find bacteriæ in the mouth of the most healthy subject, though, of course, they are most numerous in the pulpy matter upon the teeth in dirty mouths. When existing in great numbers they are capable of working great destruction.

Dr. J. W. Jay—I agree with all that has been said, especially the importance of impressing upon the mind of the patient the necessity of cleanliness. The brush is best used at night, for if the dirt is allowed to accumulate the best operations will fail. Many people will not clean the teeth at all, and think that the mere filling of the teeth is all that is necessary to preserve them; they forget all the instructions that have been given to them repeatedly, and in view of this fact it becomes the duty of the dentist to insist upon the carrying out of the measures he deems best for the patient's own good. When the gums are tender I recommend the use of a soft badger-hair brush and a solution of the white oak bark as a medicament.

Dr. Osmond—I find that a moderately stiff brush is the one that best suits the generality of cases, and it adds to the efficiency of the brush if the outside edges of it are clipped with scissors, thus preventing the bristles from getting between the teeth and the gum. It is well also that the brush be operated on a line with the teeth, instead of across them, as that manner will, in time, wear grooves across their surfaces. The unbleached bristles make the best wearing brushes, as the chemicals used in bleaching weakens the bristles and causes them to break away from their attachment, much to the disgust of the one using them. Heretofore the English made the best brushes, but lately an American firm in the East manufacture an article fully as good as any made, and, so far as I am able to judge, fully meets all the requirements.

Dr. J. Taft—Listerine, a remedy lately introduced to the notice of the profession, is, in my opinion, the best antiseptic we possess, and nothing in that line pleases me so well. It may be used full strength, or diluted, just as the judgment may deem proper. It can be made to serve a variety of purposes and with equal success. For cleansing the mouth there is nothing that will do it better, and I would say that night is probably the best time to use it. During the night, if the mouth is kept closed, there is no necessity for the use of an antiseptic, and if everybody made that practice their habit, their mouths would be the better for it. There is no doubt about it that the decay of the teeth is more rapid in the mouths of those who breathe with the mouth open, as the increased and liberal supply of oxygen greatly favors the decomposition of mucous, etc., during the hours of the night when the parts are at rest.

There is entirely too much importance placed upon the action bacteriæ are supposed to exert, as they are not prime factors in the decay of the teeth and disease of the soft parts, though they may have something to do, just what we do not yet precisely know.

Dr. J. Taft moved that the subject be passed, and that the next be taken up.

Dr. G. W. Keely moved to amend by uniting the third and fourth subjects and discussing them together, Carried.

Dr. H. A. Smith—The natural inference of the question would seem to be that we have not made much, if any, progress. I think that is true so far as filling teeth with gold is concerned, as I can cite a case where the operation was performed by Dr. J. Taft some fifteen years ago, and the work is as perfect to-day as when it was done, and I think it would be difficult to exceed that by any methods we may have discovered and practiced since. There has been, however, a decided advance made in the recognition of the histology of the teeth and in the use of plastic materials that now save many teeth that were formerly regarded as hopeless. Things have come to such a pass now-a-days that a man who makes two good operations in an hour is more useful than he who consumes an hour in making one, and makes more money. There is often too much excavating done, and the result is that students go straight for the pulp, because the softest tissue lies in that direction. I have often told them that they will do well, if, in five years, they will learn to keep away from it.

I do not agree with Dr. Taft about the bacteriæ, as I believe they have a *great deal* to do with the process of decay, etc. In very large cavities I make it a practice to leave the softened dentine immediately over the pulp, treating it with dilute carbolic acid, and afterwards washing out the cavity with alcohol or glycerine, as they have an affinity for water, and finally dry thoroughly with the warm air syringe. Then I line the cavity with melted parafine, as that is one of the most inert substances we are acquainted with. The object of the antiseptic in this course of treatment is to destroy the ferment that Dr. Miller has discovered. Formerly, we used to expose pulps by removing every trace of softened tissue, thereby admitting the air to contact with it. This I regard as bad practice, and in nine cases out of ten it is sure to end in a bad result.

Dr. G. W. Smith—I cover exposed pulps with a paste made of borax, oxide of zinc, and creasote, as this method of mixing renders the oxyphosphate inert.

Dr. Hunter—Why do you use borax with the oxide of zinc, and what advantage do you get from it?

Dr. G. W. Smith—We are all here to give our experience for the benefit of others, and this is mine. Borax is the chloride of sodium, and is inert.

Voice—Chloride of sodium is common salt.

Dr. H. A. Smith—I should like to hear from Dr. Cassidy on the subject of borax.

Dr. Cassidy—It is an antiseptic.

Dr. J. Taft—I do not think, Mr. Chairman, that it is apparent on the face of the programme that the one making it thought we had made no progress, as Dr. H. A. Smith has remarked. The idea was to take a retrospective view, and sum up what we have been doing for the last five years, and how we have done it. It is plain to all that progress *has* been made during this interval. For one thing, there is a growing realization of the importance of "maintaining the teeth and mouth in a healthy condition," as set forth in the second topic on the programme. More attention is also being directed to general hygiene for the sake of the teeth and that of the general health.

Some fifty or sixty years ago there were but very few who had any knowledge of hygiene (and they mostly kept it to themselves), but now-a-days all have more or less information concerning it, and the disposition is towards giving it more thorough care and study. There is also progress being made in the filling of teeth; more care is taken in the operation, and greater skill is constantly displayed in the handling of instruments, appliances, etc., that are continually improved upon to widen their usefulness. Besides, they are becoming more generally adopted and their use more familiar, so that we are perfecting ourselves in the use of methods upon which we base our practice. The present method of educating students renders them more capable of judging of the merits of a new thing, and in consequence they can make it more available in a shorter time. Just in proportion as we make more perfect work we make progress in saving teeth. It is my desire that every member of the profession should share the advantages that are enjoyed by the more highly educated, as that would greatly increase the influence and usefulness of our calling. The principle of conserving the softened tissue over the pulp, as detailed by Dr. H. A. Smith, is not new by any means, as it was taught twenty years or more ago, though it is now coming into more general use, for the principle is most excellent, and there cannot possibly be a better covering for the pulp than the one nature has provided. Instead of using the melted parafine as Dr. Smith suggests, I would prefer a varnish of some kind, as by that means we would avoid the heat that, in many cases, is very irritating. Progress has also been made in the treatment of sensitive dentine, as the management of it has become an easy matter by reason of the many obtunding agents, etc., that have been used with considerable success; so much so, that societies do not have to give the matter the attention they formerly did. Menthol is an agent used for treating sensitiveness, and is also used for the treatment of some of the superficial neuralgias. Probably the best way of preparing it is to make a saturated solution of the crystals in alcohol, and add a few drops of tinct. aconite; this solution obtunds sensitiveness very well.

Dr. Terry—Said that he had been experimenting for twenty years to find out a remedy for this purpose, and during that time had met with many disappointments, but that now he succeeded in making a preparation that is excellent and well worthy of a trial in the saving of exposed pulps and treatment of sensitive dentine.

Dr. Berry—The German creasote has many advantages, and is far superior to the American article.

Dr. Wright—It is wood creasote.

Dr. Berry—Yes, and it does not burn the tissues, because there is no carbolic acid in it.

Dr. H. A. Smith—I have come to the conclusion that all the foils we use for filling should come to us unannealed. Dr. Corydon Palmer was the first to tell us years ago that the first annealing is the time to use the foil, as subsequent annealings destroyed in part the cohesive power. I now buy the soft foil and anneal it just as I need it.

Dr. Sage—Can the unannealed foil be made as cohesive as that already annealed?

Dr. H. A. Smith—My conclusion is that, it *is* as cohesive, and that it can be used more rapidly, and makes just as good work as the other; and besides, inexperienced operators get along much better with it.

Dr. Van Antwerp—I have been using the unannealed, untrimmed foil; it is soft, feels like buckskin, and has no crispy harshness. I can make very good foil in my mill by rolling the gold between sheets of paper, and etching the surface with aqua regia, all traces of which, however, must be carefully removed. A second annealing of this foil renders it harsh.

Dr. J. W. Jay—I always use the unannealed foil, and always put in soft foil wherever I can do it, annealing it when I want cohesiveness.

Dr. J. Taft—I often make a gold cap to cover a cement filling when the cavity is a very large one. Melt down foil scraps, and roll out a little plate to from 28 to 33 of Stub's gauge, and then adapt and fit the cap nicely to the orifice of the cavity, soldering a pin or staple on the underside for the purpose of anchorage. Fill the cavity about full with oxyphosphate, and carefully press the cap down to its seat, holding it there until the phosphate has set. This method has the advantage of being expeditious, not very expensive, and is about as good as covering the cement with foil. Some caps that I made seven years ago are still in good working order.

Dr. G. W. Smith —How would you arrange this cap to cover fissures in the crown?

Dr. J. Taft—I only use it to cover large cavities where there are no fissures: the phosphate is better than gutta percha. The treatment of soft dentine in soft teeth, and in those of children, must be different from anything we have yet done, as it is useless to fill such teeth with gold. The reason is apparent. In sections of dentine and enamel not decayed, there will often be found imperfect tracks that frequently amount to a canal, and decay will follow down through these tracks with great rapidity. The stopping or filling of these tracks with parafine, or varnish of some kind, on the inside of the cavity, is a step in the right direction.

Dr. Sage—I was much impressed some years ago with the difficulty of saving some kinds of teeth with gold. I now use a magnifier to examine frail teeth, and always find the structure loose, with many imperfections, so that I do not fill with gold, but act upon Dr. Taft's principle of filling up the tubules with parafine or varnish.

Dr. McCampbell—I would like to hear the members give their experience in the use of Robinson's textile foil, as it is a new thing, and we don't know all about it yet.

Dr. Callahan—I use a great deal of it, especially at the cervical borders, covering it with gold. In places where there is no wear, it will become dark.

Dr. J. Taft—The tin foil made by the S. S. W. Co. is very soft, in fact, almost plastic, and should I happen to be without it when needing tin, I feel lost. The textile foil I have used somewhat; it is somewhat harder than tin, because Dr. Robinson introduced another metal to give it that property, which tin itself does not possess.

Dr. Callahan—It is strong when properly handled. I built up a bicuspid with it, putting gold enough on the outside to plate it. It is giving good service.

Dr. Emminger—I have used a good deal of Robinson's foil since its introduction. It cannot be relied upon unless it is thoroughly condensed, and it is essential to introduce it carefully.

Gold will attach itself if the surface of the foil near the orifice is not condensed too hard.

Dr. Callahan--The gold top can be pried off, but it always brings some of the tin with it.

### SECOND DAY--Afternoon Session.

Meeting opened by President Wright.

Dr. Hunter, of the Committee on Membership, presented the name of W. C. Collins for election.

On motion of Dr. J. Taft, the gentleman was elected by Secretary's ballot.

Treasurer Hunter reported that the treasury contained about fifty dollars, and presented vouchers for expenditures.

Drs. James and Irwin were appointed Auditing Committee.

On motion of Dr. Sillito, the third and fourth subjects were passed, and the fifth taken up.

Dr. Van Antwerp--There have been many valuable remedies introduced lately, among them Per-oxide of Hydrogen, which is very valuable, and I want to hear what the members have to say about it.

Dr. J. Taft--The number of available agents is continually being augmented; many of them are good, and many better than those that preceded them. In the accomplishment of our work we must, necessarily, seek for that agent which is best adapted to the case, just as we select the best instrument to perform the work in hand. I have not had an extended experience with the Per-oxide, as it is impossible to do so in three years' time. If we are seeking a disinfectant we can ascertain, in a short time, what agent will quickly break up the condition demanding its use. Anticepticism prevents decomposition; in studying the method by which it is accomplished, we find that it is done by breaking up the offensive combination and taking away one or more of its elements. The Per-oxide of Hydrogen is an efficient disinfectant, but is not so good an antiseptic. Decomposition takes place by fermentation, a small amount spreading through a large mass. Where there is a small amount of vitiated matter causing decomposition, the latter may be stopped by the removal of the former. Per-oxide of Hydrogen is simply a disinfectant.

Dr. H. A. Smith—How does the Per-oxide act?

Dr. J. Taft—By being decomposed and giving up oxygen, as it seems to me. Menthol is a crystalline preparation made from the oil of peppermint. It can be prepared in this way: Place a few crystals on a watch-glass, and dissolve them in equal parts of chloroform and tincture of aconite, applying on a pledget of cotton. I am much pleased with it, as it acts promptly, in every case, with decided success, and without any discomfort to the patient. Menthol enters, somewhat, into the preparations for relieving superficial neuralgias, something like the following: Make a saturated solution of the crystals in equal parts of chloroform and tincture of aconite; add this to an equal volume of vaseline, and mix by warming. The ointment is easily applied along the track of the nerve, with great benefit to the patient, as I have experienced upon my own person when applying it for an attack of lumbago, it operating on the sensory nerves to good effect. When applied along the track of the nerves for facial neuralgia, its effect is almost instantaneous.

Dr. Wright—Why not keep the preparation dissolved and ready for use?

Dr. J. Taft—Simply because I have not done so, thinking that the chloroform would evaporate; there may be other things, however, that will make a better combination with it. Salicylic acid is another substance that might be mentioned, though it has been in use much more than three years; it is good for some purposes, but not for others. For instance, it is a very good disinfectant, especially for treatment of roots, but it will not do to apply it when there has been a recent inflammation about the root of the tooth, as it only tends to irritate and increase the difficulty. The best results obtained from it are in the treatment of low chronic forms of inflammation. Iodoform is used, and is good for the same purposes as salicylic acid. Resorcin is a good disinfectant, and acts in this way very much like salicylic acid and iodoform, though it has an advantage over the latter in being preferable, because it has not the disagreeable and pungent odor.

Dr. J. W. Jay--Do you, or have you, used the oil of eucalyptus?

Dr. J. Taft--Yes; but principally as an antiseptic; it is, also, a gentle stimulant; it has no advantages over other agents in use, and I doubt if it is any better than thymol.

Dr. Hoff—How about listerine, the new preparation lately introduced?

Dr. J. Taft—It is a good disinfectant for the dentist to use about the office, generally, and is not expensive. If a patient's mouth has an unbearable odor, make him wash out his mouth with it. It has taken the place of many agents heretofore used for a variety of purposes. A teaspoonful, in some water, does very well to wash the teeth at night. It is, also, an excellent deodorizer.

Dr. Wright—Does it accomplish the purpose as well as Per manganate of Potash?"

Dr. J. Taft—Better, probably; at least quite as well, and, besides, does not stain the parts. It is not, as some may think, a nostrum, the formula being printed on every bottle.

Dr. H. A. Smith—I do not place the same value on salicylic acid for the reason that it destroys tooth tissue, acting on the dentine as a solvent. In answer to a question Dr. Taft stated that the per oxide of hydrogen acts by giving up oxygen; that is true, and is, moreover, all we know about it. Many years ago it was used to bleach teeth, and also as a deodorizer. Koch and others place their reliance upon one agent, when either a disinfectant or an antiseptic is required. Listerine is a compound or mixture of many ingredients, and, as we are not yet very familiar with it, we must wait and see the results obtained from its use. I give the preference to per manganate of potash; it is a powerful disinfectant, but I cannot say whether it is an antiseptic or not. Carbolic acid stands at the head to-day as a germicide; it is an anti-ferment and a good disinfectant. If you want to sterilize the extract of beef or arrest fermentation use carbolic acid. Dr. James uses, or did use, a pretty strong solution of the bi-chloride of mercury; and I, for one, would like to have him tell us about it.

Dr. James—I did use it in one case, because it needed it; the result was very satisfactory, and the tooth is still good.

Voice—What was the strength of your solution?

Dr. James—One grain to the ounce; the application of it is best made on a broach, introduced into the canal. There has been no evil result so far in the case I used it.

Dr. Van Antwerp—Physicians that use it do not make it near so strong, the proportion being about 1 part in 800.

Dr. H. A. Smith—The smallest portion of the bi-chloride is a sure shot on both fermentation and germs, and I greatly admire Dr. James' courage in using it.

Dr. Van Antwerp—Medical practitioners are now trying to destroy tubercle bacilli by the inhalation of the bi-chloride.

Dr. H. A. Smith—It seems to me that it is possible, and not improbable, that in an amalgam filling a salt of mercury may be formed something like the bi-chloride, which, saturating the tooth and entering the tubules, arrests caries accordingly. This is a little theory of my own, and based merely on therapeutical action.

Dr. J. Taft—The vapor of mercury is exceedingly volatile and penetrating. On this account the vapor does, in many instances, penetrate the dentine, and in some cases throughout. The vapor having passed into the tubuli oxidizes right there, and the particles of oxide being larger and unable to get out stop up the tubules, thus acting as a shield. This process is very likely to occur in soft teeth, as the vapor very readily passes in as I have described. If the teeth are dense no vapor will pass in. Amalgam can be so prepared that but little vapor of mercury will exhale from it.

Dr. H. A. Smith—As we also find chlorine in the mouth, why should it not unite with the oxygen also present?

Dr. J. Taft—Oxygen coming from decomposition in the mouth unites with the mercury to form the oxide, and circumstances favor the formation of the oxide, rather than the bi-chloride; if they did not we would have the most powerful argument against the use of amalgam, which would then be almost criminal.

There is a distinction between disinfectant and antiseptic. A *disinfectant* acts by breaking up the offensive compound; takes away from it one of its constituents and leaves a residuum of inert material; or it may form new compounds that are innoxious.

An *antiseptic* is a different thing; it sets up an influence in a substance or material about to decompose and prevents or prohibits the decomposition.

Dr. H. A. Smith---What makes or causes the decomposition?

Dr. J. Taft---When life force is destroyed decomposition sets in, oxygen being the great agent occasioning the decomposition. Any agent that will prevent this is called an antiseptic. If the air is excluded the process of decomposition will not go on, as the presence of oxygen is usually necessary to carry it out. Any agent that makes a cover excluding oxygen and living organisms constitutes an antiseptic; carbolic acid acts as such by coagulating albumen.

Dr. H. A. Smith---Excuse me, Mr. President, for "harping on my daughter," but I must stick to my amalgam filling. We have chlorine found in every mouth, and I do not see any reason why it should not unite with free mercury and form the bi.chloride. An amalgam that blackens reasonably well is, according to my theory, a good one; the blackening may be due to the presence of silver or copper in the amalgam.

Dr. J. Taft---The coloring matter found in the dentine of such cases may be due to the sulphuret of copper or mercury or the oxide of silver; but I think it is owing to the oxidation of the volatile vapor of mercury that has passed into the tubules. The oxide of silver is too gross a substance to be a cause. The chlorine will attack calcareous matter every time in preference to mercury when the two are presented together to its action.

Dr. Sage—I doubt if the per-oxide of hydrogen is permanently effective. It merely acts as a policeman, and drives out the offensive matter for the time being.

Dr. Sillito--I recently read of some cases in a medical journal relating to the treatment of ulcers with the per-oxide, followed by granulation.

Dr. J. Taft—The gaseous bubbling that is noticed when the per-oxide is used is owing to the liberation of oxygen, and shows the action to be prompt.

Dr. Hoff—I have used the per-oxide for six months to the exclusion of creasote, and like it extremely well. It is very easily applied, and can be done so freely without any danger of cauterization of parts it may happen to flow upon. It has proved a perfect success with me. One case in particular for which I used it demonstrates its efficacy; a tooth had been affected with an abscess of six years standing, it was loose and the process pretty well gone. I opened the tooth, removed the *debris* of the pulp, and used the per-oxide on cotton. I also found a cavity or pocket in the process which I injected. The tooth was subsequently filled with oxy-phosphate, and, so far, has behaved itself satisfactorily.

Dr. Hunter—I would like to know if any one has used the capiscum bags introduced by Dr. Flagg, and what their experience has been?

No reply.

On motion the subject was passed, when Dr. Howe, Chairman of the Committee on Resolutions on the death of Prof. Buckingham, submitted the following report, which was adopted:

IN MEMORIAM.

This Association desires to place on record its sense of the great loss which the dental profession has sustained by the death of Prof. T. L. Buckingham, of Philadelphia, Penn.

We may not hope, by any form of words, to adequately express the value of his faithful and life-long devotion to the educational and practical interests of the whole profession, or of his most estimable character as a man, but we offer this heartfelt tribute in token of sincere appreciation of what he has wrought for us. We wish, also, to convey to his bereaved family our sympathy with them in this time of their sore affliction and irreparable loss.

The Secretary is instructed to transmit to the family a copy of this record in behalf of the Mississippi Valley Dental Association, in annual session at Cincinnati, Ohio, March 6, 1884.

W. S. Howe,<br>

G. W. Smith, } *Committee.*<br>

J. Taft,

The sixth and last subject on the programme was presented for discussion, Drs. G. W. Smith, Clyde, Berry, and others, making a few remarks. The subject was not fully discussed, by any means, because the day was drawing to a close, and as the programme had been gone over, with the exception of the last topic, it was decided to pass it, go into the election of officers, and adjourn.

The officers elected for the following year are as follows:

Wm. Van Antwerp, President, Mt. Sterling, Ky.

F. W. Sage, 1st Vice-President, Cincinnati, O.

A. F. Emminger, 2nd Vice-President, Columbus, O.

A. G. Rose, Recording Secretary, Cincinnati, O.

F. A. Hunter, Treasurer, Cincinnati, O.

A. Berry, Corresponding Secretary, Cincinnati, O.

The following standing committees were appointed by the President:

Exec: E. G. Betty, C. R. Taft, N. S. Hoff.

Memb; F. A. Hunter, J. S. Cassidy, A. G. Rose.

Pub: J. Taft, J. M. Clyde, O. N. Heise.

Ethics: G. W. Keely, H. A. Smith, C. M. Wright.

In the absence of the President-elect, the first Vice-President, Dr. Sage, was conducted to the Chair.

After the payment of bills for printing, postage, etc., and the exchange of many pleasantries, the Association adjourned to meet again the first Wednesday in March, 1885, at 10 A. M.

